# *Abcc6* deficiency prevents rhabdomyolysis-induced acute kidney injury

**DOI:** 10.1038/s41598-023-47894-z

**Published:** 2023-12-06

**Authors:** Audrey Casemayou, Julie Belliere, Emmanuel Letavernier, Eloïse Colliou, Hélène El Hachem, Jeremy Zarowski, Dominique Bazin, Clément Kounde, Alexis Piedrafita, Guylène Feuillet, Joost P. Schanstra, Stanislas Faguer

**Affiliations:** 1 Department of Nephrology and Organ Transplantation, Reference Centre for Rare Kidney Diseases (SORARE), French Intensive Care Renal Network (FIRN), University Hospital of Toulouse, 1, Avenue du Pr. Jean Poulhes, 31059 Toulouse Cedex, France; 2 National Institute of Health and Medical Research (INSERM), Institute of Metabolic and Cardiovascular Diseases (I2MC), UMR 1297, Rangueil Hospital, 31000 Toulouse, France; 3 University Toulouse-3, 31000 Toulouse, France; 4 National Institute of Health and Medical Research, (INSERM) UMR S 1155, Tenon Hospital, 75020 Paris, France; 5 Sorbonne University, 75020 Paris, France; 6 Department of Physiology, Tenon Hospital, Assistance Publique – Hôpitaux de Paris, 75020 Paris, France; 7 Laboratory of Chemistry and Physics, CNRS UMR 8000, University Paris XI, 91405 Orsay, France

**Keywords:** Acute kidney injury, Acute inflammation

## Abstract

Rhabdomyolysis is a risk factor for acute kidney injury, transition towards chronic kidney disease, and death. The role of calcium phosphate deposits in the mechanisms of rhabdomyolysis-induced acute kidney injury (RAKI) is still unclear. Better insight of the role calcium in RAKI could lead to new therapeutic avenues. Here, we show in a mice model of RAKI that calcium phosphate deposits were frequent in the kidney (hydroxyapatite) and partly correlated with the severity of the kidney injury. However, the intensity of deposits was highly heterogeneous between mice. Treatment with sodium chloride, sodium bicarbonate or inorganic pyrophosphate (PPi; an inhibitor of the calcium phosphate crystallization), or combinations thereof, did not improve kidney outcomes and hydroxyapatite deposition during RAKI. Unexpectedly, *Abcc6* knockout mice (*ko*), characterized by PPi deficiency, developed less severe RAKI despite similar rhabdomyolysis severity, and had similar hydroxyapatite deposition suggesting alternative mechanisms. This improved kidney outcome at day 2 translated to a trend in improved glomerular filtration rate at month 2 in *Abcc6*^*-/-*^mice and to significantly less interstitial fibrosis. In addition, whereas the pattern of infiltrating cells at day 2 was similar between *wt* and *ko* mice, kidneys of *Abcc6*^-/-^ mice were characterized by more CD19^+^ B-cells, less CD3^+^ T-cells and a lower R1/R2 macrophage ratio at month 2. In summary, kidney calcium phosphate deposits are frequent in RAKI but hydration with sodium bicarbonate or sodium chloride does not modify the kidney outcome. Blocking ABCC6 emerges as a new option to prevent RAKI and subsequent transition toward kidney fibrosis.

## Introduction

Rhabdomyolysis is a life-threatening disorder with a high risk of the development of severe acute kidney injury (AKI), hyperkalemia and metabolic acidosis^1,2^. Beyond earthquakes and wars that are archetypical situations associated with severe rhabdomyolysis owing to crush and limb ischemia, several critical conditions can lead to rhabdomyolysis including infection, drugs and envenomation^3^. To date, the treatment of rhabdomyolysis is mostly symptomatic and aims to reverse the causes of rhabdomyolysis, prevent additional kidney injuries (hypovolemia, shock, drugs) and treat metabolic complications (hydration, dialysis).

Rhabdomyolysis-induced AKI (RAKI) is a complex disease that combines renal hypoperfusion (hypovolemic shock related to muscle edema and vasoconstriction related to myoglobin-dependent nitric oxide depletion), direct toxicity to proximal tubular cells (myoglobin and uric acid-induced cell necrosis), intra-renal inflammation (macrophage infiltration, complement and NLRP3 inflammasome activation) and distal tubule obstruction (myoglobin – uromodulin complex)^1,4–6^. In addition to the release of high load of nephrotoxic molecules (uric acid and myoglobin) in the circulation, rhabdomyolysis and other cell lysis syndrome are also accompanied by severe hyperphosphatemia. Even though pathological data are scarce, acute hyperphosphatemia may directly induce AKI. This was reported in acute phosphate nephropathy induced by oral sodium phosphate bowel solutions^7^, and in some cases of RAKI^8^. Recently, we have reported that serum phosphorus at hospital admission was correlated with the risk to develop severe KDIGO stage 2–3 AKI, independently of the other markers of rhabdomyolysis severity^2^. Therefore, targeting phosphorus load in RAKI might be a valuable treatment option. However, before proposing a potential new treatment in RAKI, a better characterization of the role of calcium phosphate deposition within kidneys in AKI is required.

The goals of current treatment strategies to prevent or reverse RAKI are to avoid hypovolemia and increase the urinary elimination of myoglobin and potassium^9^. Alkalinization of urine with, for example, sodium bicarbonate may prevent the development of myoglobin-uromodulin complex^10^, but also increase the rate of calcium phosphate deposition in alkaline conditions. Therefore, treatment strategies currently mainly recommend intensive hydration with sodium chloride solute, even if in turn this latter may theoretically decrease the urinary pH and subsequently increase myoglobin precipitation within tubules. However, the available data in animal models of RAKI or in humans are scarce, precluding treatment optimization. Modulating the risk of calcium phosphate crystallization with inorganic pyrophosphate (PPi) may be a therapeutic option, but has not been verified in preclinical models yet. Injection of PPi may reduce the rate of calcium phosphate crystallization^11^, whereas *Abcc6* invalidation and subsequent PPI deficiency may increase the risk of vascular and kidney calcium phosphate deposits^12^.

In this interventional study in mouse, we aimed to characterize the role of phosphorus deposition within the kidney during RAKI, identify the optimal hydration solute and test the ability of modulating PPi levels to prevent RAKI.

## Methods

### Animal studies

All experimental procedures were performed in accordance with institutional guidelines for ani-mal studies and were approved by the national ethics committee (APAFIS: approval number N°202,012,041,500,456). Rhabdomyolysis was induced in mildly sedated (isoflurane) 8–10-week-old male C57Bl6 mice (Charles River Laboratories, France) by intramuscular injection in each thigh caudal muscle with 7.5 ml/kg 50% glycerol (VWR International, Radnor, Pennsylvania, USA), as previously reported^6^.

According to study groups, mice received intraperitoneal injection of sodium chloride (0.2 mL), sodium bicarbonate 4.2% (0.2 mL), or PPi (12.5 mg/kg diluted in 0.2 mL of sodium chloride).

*Abcc6*^-/-^ mice, formerly designated as *Abcc6*^tm1Aabb^ were generated on a 129/Ola background, and then backcrossed into a C57Bl/6 J background more than ten times. These mice were maintained at the INSERM U1291 facilities (Paris, France). For experiments (Fig. 4 and Supplementary Figs. 1–3), *Abcc6*^-/-^ mice were compared to *Abcc6* wt littermates.

To obtain mice expressing the GFP marker specifically in the proximal tubule cells, we crossed the KAP2-iCre mouse line (Jackson n°008,781), which expressed a CRE-recombinase in proximal tubule cells in male mice (spontaneously) or in female mice (after exposition to testosterone), with a mouse line harboring a GFP gene flanked by *LoxP* sequences.

Mice were sacrificed with a sublethal injection of Dolethal (0.182 mg per g of mice) and transcardially perfused with 2 ml PBS. For histology, kidneys were either fixed in 4% PFA or in Carnoy solution. For mRNA analysis, kidneys were snap-frozen in liquid nitrogen. Studies in animals were reported according with ARRIVE guidelines.

### Biological tests

Blood was collected from the mouse tail vein in EDTA tubes at 6 h, 2 days or 20 days after glycerol injection and was centrifuged at 2000 rpm for 5 min to separate plasma. Plasma BUN, phosphorus and CPK were analyzed on a Pentra 400 analyzer (Horiba Medical). For GFR determination, FITC-sinistrin half-life was measured. In short, under anesthesia, a transcutaneous device (MediBeacon) was attached to the depilated skin on the back of mice using a double-sided adhesive patch. Transcutaneous measurement started with background reading one to three minutes before FITC-Sinistrin was administered intravenously (7 mg/100 g body weight). Animals were allowed to fully recover and move freely until transcutaneous measurement was stopped after 60 min.

### Quantitative polymerase chain reaction

mRNA was isolated from frozen kidneys using the RNeasy Plus Purification kit (Qiagen) and 500 ng were reverse-transcribed using High-Capacity cDNA reverse Transcriptase (Applied Biosystem) or not (RT–) to exclude genomic DNA contamination. Real-time quantitative PCR was performed using the ONE Green PCR master mix (Ozyme). Analysis of GAPDH mRNA was performed to normalize gene expression using the 2^-ΔΔCt^ method.

### Kidney pathology

Formalin or Carnoy fixed kidneys were embedded in paraffin, sectioned in 4 μm thick slices and stained with filtered Sirius red 1% (BDH laboratories), Masson’s Trichrome, and Von Kossa staining. For immunohistochemistry, rabbit anti-myoglobin (1/100, Invitrogen, Waltham, United States) and rat anti-uromodulin (1/100, Fisher Scientific, Hampton, United States) antibodies were incubated for 1 h at room temperature. Following this, the specimens were washed twice with TBS 0.1% Tween 20 and incubated with Histofine simple stain MAX-PO (Nichirei) for 30 min. For detection of signals the Dako Envision system (K4010; Dako) was used. Finally, sections were counterstained with hematoxylin, dehydrated and mounted. Sections were scanned using a Nanozoomer 2.0 RS (Hamamatsu) and analyzed with image J software.

### Field emission-scanning electron microscopy (FE-SEM)

Tissue Sects. (4 µm) were investigated with a Zeiss SUPRA^TM^55VP field emission-scanning electron microscope (FE-SEM). Measurements were performed at a low voltage (1.4 keV) and without the usual deposits of carbon at the surface of the sample.

### FTIR micro-spectroscopy

Microcalcifications were characterised using Fourier Transform InfraRed microspectroscopy (µ-FTIR). Tissue Sects. (4-µm) were deposited on low-emission microscope slides (MirrIR, Keveley Technologies). FTIR analysis was performed in serial sections adjacent to tissue sections stained with Yasue technique. FTIR hyperspectral images were recorded with a Spectrum spotlight 400 FT-IR imaging system (Perkin Elmer Life Sciences), with a spatial resolution of 6.25 µm and a spectral resolution of 8 cm^−1^. The spectra were recorded in the 4000–700 cm^−1^ mid-InfraRed range. Each spectral image covering a substantial part of the tissue, consisted of about 40,000 spectra.

### Fluorescence-activated cell sorting (FACS)

Kidneys were decapsulated, minced, and incubated with collagenase (2 mg/mL, Sigma-Aldrich) and DNAse (1KU/*µ*l, Qiagen). After red blood cell lysis, cells were passed through a 40-*µ*m mesh and stained with Viobility (Biolegend). Then cells were incubated with anti-CD16/32 (Biolegend) and stained with anti-CD45-PE violet 770 (BD), anti-CD11b-PE violet 615 (BD), anti-F4/80-APC (BD), anti-Ly6C-PerCP violet 700 (BD), anti-Ly6G Alexa Fluor700 (Biolegend), anti-CD3-PE (BD), anti-CD4-APC Violet 770 (BD), anti-CD8a viogreen 520 (BD), anti-CD19 viobright 515 (BD), and anti-NK 1.1-Billant violet 605 (Biolegend). A known quantity of Countbright beads (Molecular Probes) was added. Acquisition was performed on a BD LSR-Fortessa cytometer. Standard analyses were performed on FACS Diva Software (Becton Dickinson, Franklin Lakes, NJ). Gates were made as follows: CD19^+^ B cells: CD45^+^ Viobility^-^ CD11b^-^ F4/80^-^ CD3^-^ CD19^+^ ; CD3^+^ T cells: CD45^+^ Viobility^-^ CD11b^-^ F4/80^-^ CD3^+^; CD3^+^ CD8^+^ T cells: CD45^+^ Viobility^-^ CD11b^-^ F4/80^-^ CD3^+^ CD8^+^; CD3^+^ CD4^+^ T cells: CD45^+^ Viobility^-^ CD11b^-^ F4/80^-^ CD3^+^ CD4^+^; R0: CD45^+^ Viobility^-^ CD11b^+^ F4/80^-^ ; R1: CD45^+^ Viobility^-^ CD11b^+^ F4/80^low^; R2: CD45^+^ Viobility^-^ CD11b^+^ F4/80^+^

### Statistical analysis

Quantitative variables are shown as mean ± SEM and compared with the Mann–Whitney test (2 groups) or Kruskal–Wallis test, as appropriate. Qualitative variables are shown as number and percentages and compared with the Fischer exact test. Correlation between BUN and calcium phosphate deposits was assessed with the Spearman test. A p-value below 0.05 was considered significant. The datasets used and/or analyzed during the current study are available from the corresponding author on reasonable request.

## Results

### RAKI is characterized by hydroxyapatite deposition in kidneys

**Figure 1:**
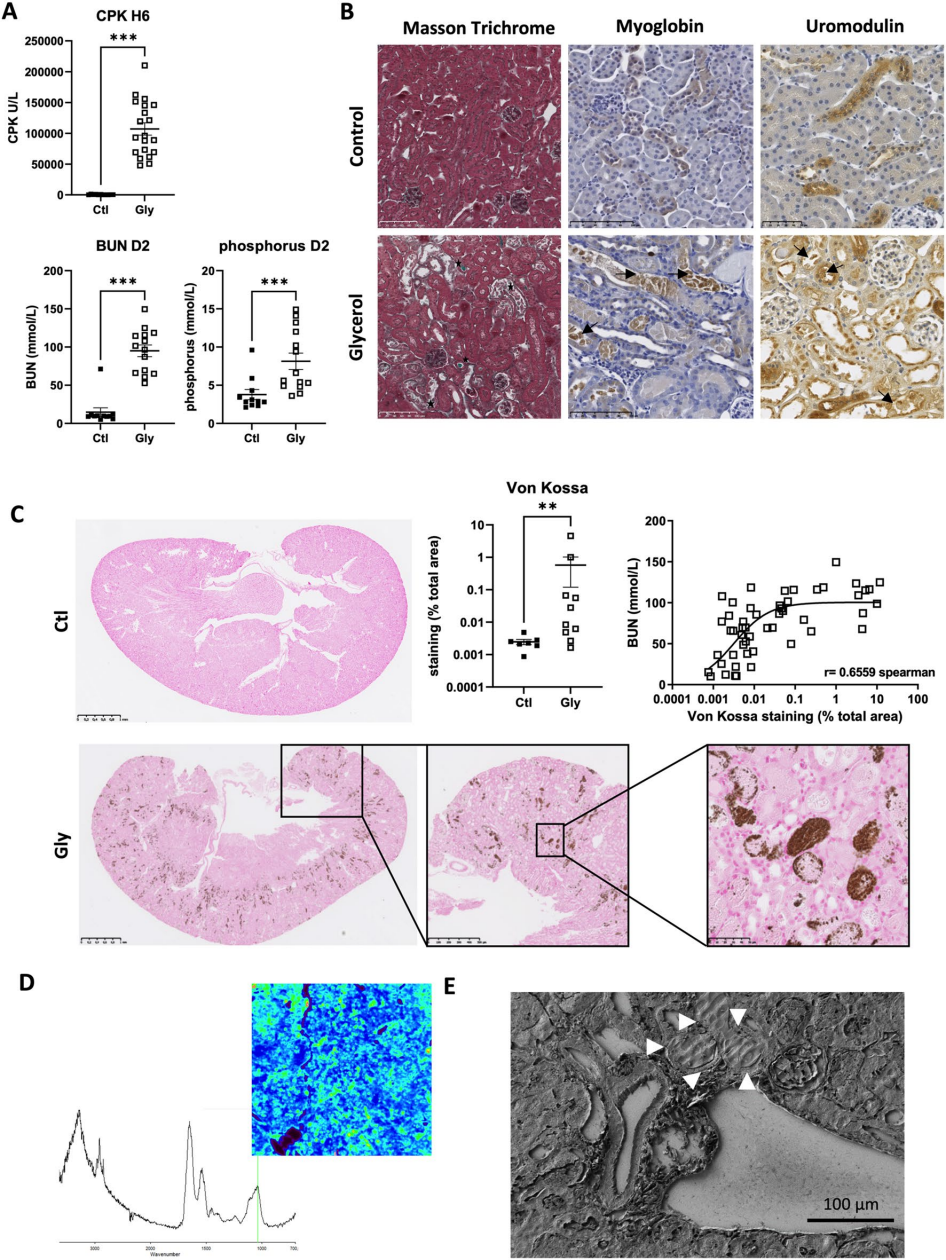
Hydroxyapatite deposition within kidneys after glycerol-induced rhabdomyolysis. (**A**) Plasma levels of creatine phosphokinase (CPK) at 6 h (H6) and blood urea nitrogen (BUN) and phosphorus at day 2 (D2) after glycerol (Gly) or NaCl (Ctl) injection in each thigh caudal muscle. Comparisons were made with the Mann–Whitney test (n = 11–15 mice per group). (**B**) Representative images of Masson Trichrome, myoglobin and uromodulin staining at day 2 after glycerol (Gly) or NaCl (Control) injection (arrows show the positive staining for uromodolin and myoglobin in tubular casts, tubular cells and interstitium). (**C**) Representative images and quantification of calcium phosphorus deposits (Von Kossa staining). Comparisons were made with the Mann–Whitney test (n = 11–15 mice per group). Correlation between Von Kossa staining and BUN at day 2 (Spearman’ test). (**D**) Plug reflectance FTIR cartography and reflectance spectrum showing a peak at 1035 cm^−1^ specific for apatite (green bar) and a smooth and rounded peak between 1035 and 1100 cm^−1^ revealing the presence of amorphous calcium phosphate with a significant carbonate rate (peaks at 875 cm^−1^ and 1420 cm^−1^). (**E**) Scanning electron microscopy showing the presence of dense tubular plugs made of calcium phosphate (arrows). Values are expressed as mean ± SEM. *p < 0.05, **p < 0.01, ***p < 0.001, compared with the control group.

Following glycerol injection, C57BL/6 J mice developed rhabdomyolysis (CPK: 107,030 ± 44,812 U/L at 6 h) and AKI (BUN: 95 ± 28 mmol/L at day 2) with frank hyperphosphoremia (phosphorus 8 ± 4 mmol/L at day 2) (Fig. 1A). Kidney pathology confirmed tubular injury and the formation of numerous casts within distal tubules (Fig. 1B, stars). These casts were positively stained with myoglobin and uromodulin (arrows). Myoglobin staining was also positive in some tubular cells (Fig. 1B, arrows).

Von Kossa staining, infra-red spirometry and electron microscopy showed multiple deposits of calcium phosphate within kidneys (hydroxyapatite) (Fig. 1C–E). Intensity of the deposits was highly heterogeneous between mice and deposits were located within the tubular lumen (necrotic cells or casts) and cytoplasm of proximal tubule cells but also in the interstitium. The extent of deposits was log-linearly correlated with the severity of AKI (Fig. 1C).

### Sodium chloride, sodium bicarbonate or PPi treatment do not prevent AKI and intra kidney calcium phosphate deposition after rhabdomyolysis onset

**Figure 2:**
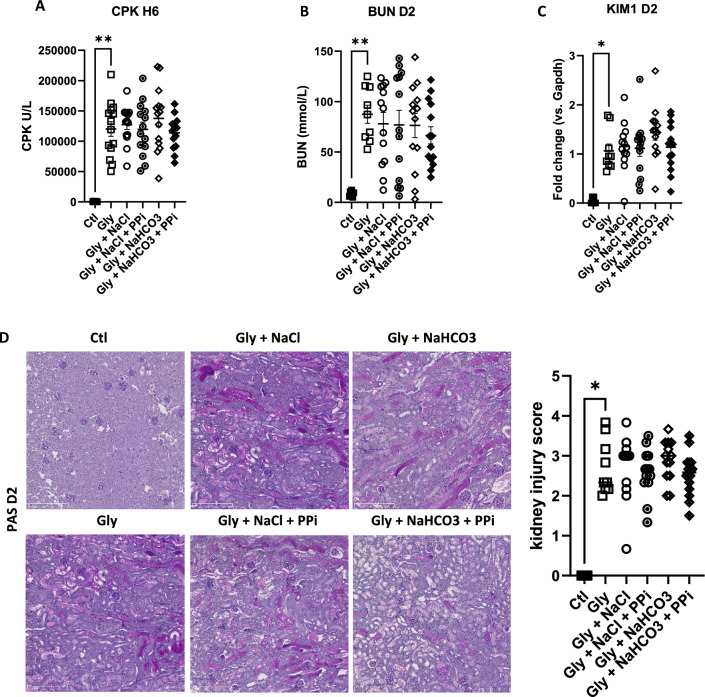
Sodium chloride, sodium bicarbonate and inorganic pyrophosphate (PPi) do not modify kidney outcomes of rhabdomyolysis-induced acute kidney injury. (**A, B**) Plasma levels of creatinine phosphokinases (CPK) at 6 h (H6) (**A**) and blood urea nitrogen (BUN) at day 2 (D2) (**B**) after rhabdomyolysis (Gly) and rehydration with sodium chloride (NaCl), sodium bicarbonate (NaHCO3), NaCl + PPi, NaHCO3 + PPi or no rehydration. *Ctl*, control (no rhabdomyolysis) (**C**) mRNA expression of KIM-1, a marker of kidney injury, in kidneys of mice with RAKI, according to rehydration protocols. (**D**) Representative images of RAKI (Schiff periodic acid staining) according to rehydration protocols and comparison of the injury scores. Values are expressed as mean ± SEM. *p < 0.05, **p < 0.01, compared with the glycerol group.

**Figure 3:**
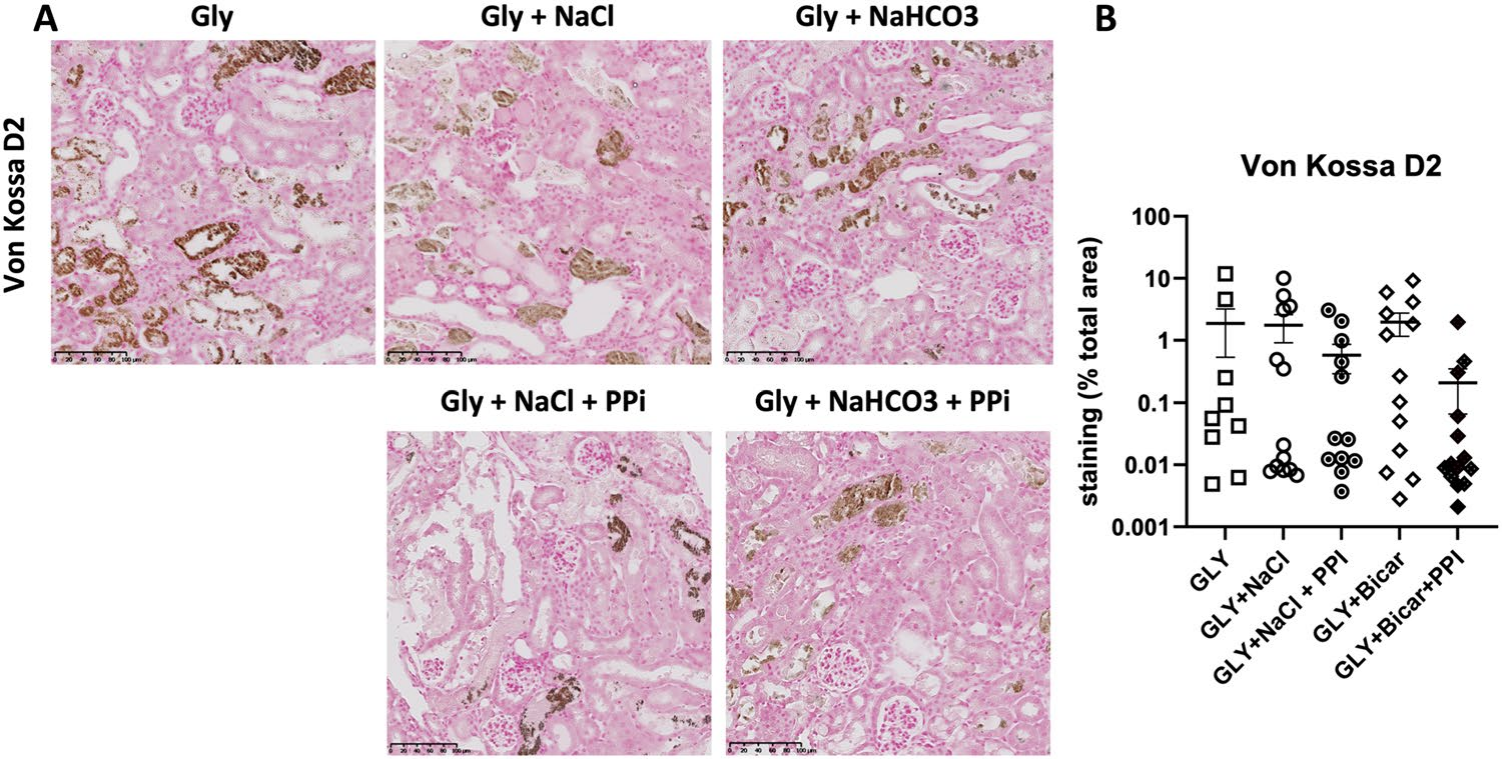
Sodium chloride, sodium bicarbonate and inorganic pyrophosphate do not modify calcium phosphate deposition during rhabdomyolysis-induced acute kidney injury. (**A**) Representative images of hydroxyapatite deposition at day 2 of RAKI, according to rehydration protocols (sodium chloride (NaCl), sodium bicarbonate (NaHCO3), inorganic pyrophosphate (PPi)). (**B**) Correlation of hydroxyapatite deposition (Von Kossa staining) and blood urea nitrogen (BUN) at day 2.

We first evaluated in the RAKI model the effect of currently used AKI preventive treatments in the clinic. To be as close as possible to the clinical situation we treated animals 6 h after rhabdomyolysis induction. Despite similar severity of rhabdomyolysis (blood levels of CPK) (Fig. 2A), treatment with sodium chloride, sodium bicarbonate or PPi, or combinations thereof, did not improve kidney function as assessed by blood urinary nitrogen (BUN) concentrations (Fig. 2B), *Kim1* mRNA expression (Fig. 2C), kidney histology (Fig. 2D) and intra kidney calcium phosphate deposition (Fig. 3A,B), 2 days after rhabdomyolysis.

### Mice with Abcc6 deletion are protected from RAKI

These surprising observations of the total absence of a benefit of these different treatments, employed in the clinic, on both severity and calcium phosphate deposition in this RAKI model sparked our interest to better understand the role of calcium phosphate deposition in RAKI. With this aim we focused our attention on crystallization inhibition by PPi by using a mouse model of PPi deficiency related to deletion of *Abcc6*, a gene coding for an ATP transporter^12^.

As a first step, using quantitative PCR performed in FACS-sorted tubular and immune kidney cells, we confirmed the expression of *Abcc6* in proximal tubule cells but also identified *Abcc6* mRNA in CD45 + immune kidney cells (Supplementary Fig. 1). In *wt* mice, kidney expression of *Abcc6* was significantly reduced at day 2 and remained low at day 20, despite BUN normalization (Supplemental Fig. 2).

**Figure 4:**
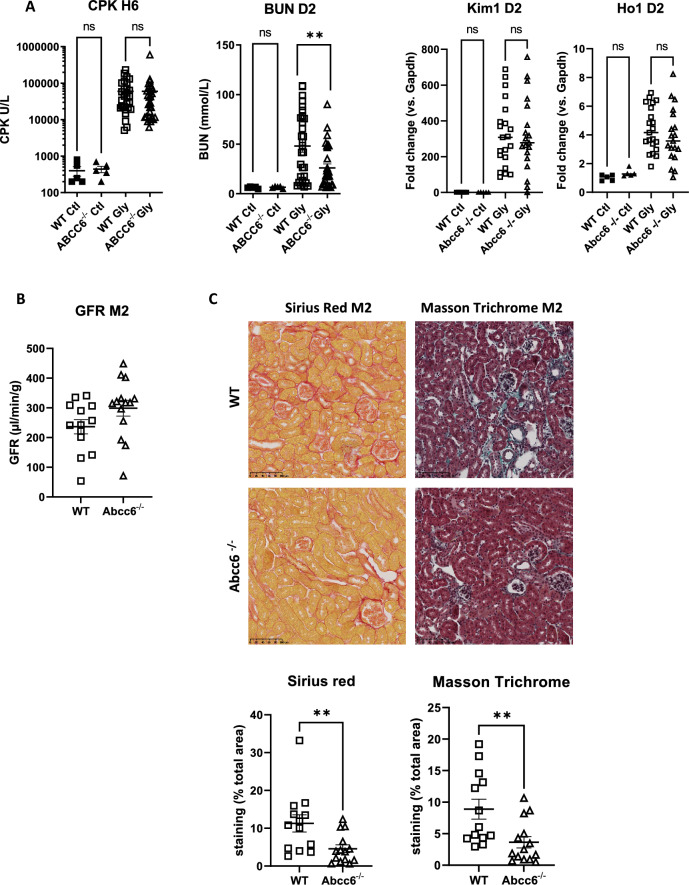
ABCC6 deficiency prevents rhabdomyolysis-induced acute kidney injury. (**A**) Plasma levels of creatine phosphokinase (CPK) at 6 h (H6), blood urea nitrogen (BUN) at day 2 (D2) and mRNA kidney expression of KIM1 and HO1 after glycerol injection. Comparisons were made with the Mann–Whitney test (n = 11–23 mice per group) and outliers were excluded by a Grubbs test. (**B**) Measured glomerular filtration rate (GFR; Sinistrin-FITC) at 2 months. (**C**) Representative images of Sirius Red and Masson Trichrome staining at month 2 after glycerol injection in wild-type (WT) and *Abcc6*^-/-^ mice. Values are expressed as mean ± SEM. *p < 0.05, ** *p* < 0.01, compared to WT.

Unexpectedly, as shown in Fig. 4A, *Abcc6*^*-/-*^ mice developed less severe RAKI despite similar rhabdomyolysis severity. At day 2, BUN was lower in *Abcc6*^*-/-*^mice accompanied by reduced renal *Ho-1* mRNA expression and a trend in lesser *Kim-1* expression. In these experiments, both *wt* and *ko* mice had very low and similar levels of calcium phosphorus deposition (Supplementary Fig. 3). Thus, ABCC6 deficiency was not associated with increased calcium phosphorus deposition, suggesting RAKI mechanisms largely independent of calcium phosphate deposition.

The better kidney outcome at day 2 translated to a trend in better glomerular filtration rate at month 2 in *Abcc6*^*-/-*^mice (mean GFR: 299 ± 100 vs. 236 ± 86, *p* = 0.07) (Fig. 4B). Interstitial fibrosis, as quantified by Masson trichrome and Sirius red staining was significantly reduced in *Abcc6*^-/-^ mice (Fig. 4C).

### Abcc6 deletion modulates kidney immune cell infiltration after RAKI

**Figure 5:**
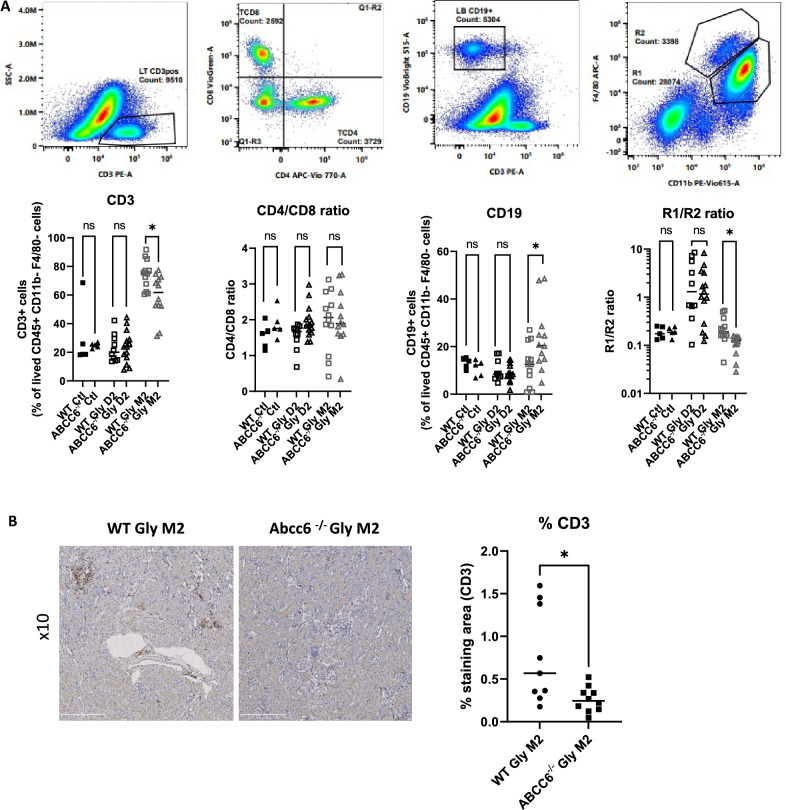
ABCC6 deficiency modulates the kidney immune response to rhabdomyolysis-induced acute kidney injury. (**A**) Immune cells pattern within kidneys at day 2 and 2 months after glycerol injection. Values are expressed as mean ± SEM. * *p* < 0.05, *** *p* < 0.001, compared to WT. (**B**) CD3^+^ immunostaining on kidney 2 months after glycerol injection.

With an absence of an effect on calcium phosphorus deposition in *Abcc6*^*-/-*^ mice, we started to look for alternative hypotheses to decipher how ABCC6 could modulate the kidney outcome after RAKI. Therefore, we analyzed the immune cell infiltration pattern at day 2 and month 2 following rhabdomyolysis. As shown in Fig. 5A, analysis of the alive CD45^+^ cells from the kidneys at day 2 showed an unmodified proportion of CD8^+^ and CD4^+^ CD3^+^ T-cells, R1 and R2 macrophages, as well as CD19^+^ B-cells in *ko* mice compared to *wt* mice. In contrast, at month 2, kidneys of *Abcc6*^-/-^ mice were characterized by more CD19 + B-cells, less CD3^+^ T-cells (with similar CD4^+^/CD8^+^ ratio) and a lower R1/R2 macrophage ratio (Fig. 5A). The decrease of CD3 + T-cells infiltration at month 2 was confirmed with anti-CD3 + immunostaining (Fig. 5B).

## Discussion

Kidney toxicity due to the risk of acute calcium phosphate deposition is suspected when calcium salts are perfused to patients with high serum levels of phosphorus (for instance, in patients with cell lysis syndromes)^1^. This hypothesis is supported by the observation of calcium phosphate kidney deposits in cell lysis syndrome^13^ and the risk of acute phosphate nephropathy after massive phosphate-salt ingestion for colonic preparation^7^. However, whether targeting calcium phosphate deposition may modulate the outcome of RAKI remains elusive. Here we showed that, whereas calcium phosphate deposition in the kidney is a feature of RAKI, administration of sodium bicarbonate, sodium chloride or PPi, or combinations thereof, did not translate in better kidney outcome, suggesting that calcium phosphate passively deposit in injured kidney tissues rather than fueling the injury process. Moreover, whereas calcium phosphate deposition was recurrently observed in C57Bl6 mice from Charles River laboratories (France), no or very few deposition was observed in mice with *Abcc6* invalidation or their littermate (Supplementary Fig. 3), also suggesting calcium phosphorus deposition is not a major trigger of RAKI. If confirmed, serum phosphorus should thus no longer be considered as a potential therapeutic target in lysis syndromes.

Alternative therapies not targeting phosphorus are thus mandatory to improve the kidney and overall outcomes of patients developing rhabdomyolysis. Interestingly, while we hypothesized *Abcc6*^-/-^ mice would be more prone to develop severe RAKI and subsequent kidney fibrosis, we observed a better kidney outcome in these mice. ABCC6 (ATP binding cassette subfamily C member 6) is an ATP-dependent transporter mainly expressed in hepatocytes, but also at the basolateral membrane of kidney proximal tubules cells where its role is unknown^14^. *Abcc6* is not expressed in other parts of the kidney. Inherited ABCC6 deficiency is associated with the development of *pseudoxanthum elasticum*, a rare inherited disease characterized by a PPi deficit, vascular calcification, and urolithiasis^12,15^. ABCC6 contributes to the outflow of ATP from cells to the extracellular environment, where it is hydrolyzed to AMP and PPi by ectonucleotidase ENPP1. AMP is subsequently converted to adenosine and phosphate by the ecto-5’-nucleotidase CD73^16^. AMP and adenosine have various purinergic signaling-dependent effects including macrophage activation, vascular contractility, and cytoskeletal rearrangement in epithelial cells^16^. Independently of its role in PPi metabolism and circulating levels, deletion of *Abcc6* may thus theoretically prevent several key pathogenic mechanisms of RAKI, including rhabdomyolysis-induced macrophage-dependent kidney inflammation^6^, myoglobin-induced nitric oxide-dependent vasoconstriction^17^ and ATP depletion-induced tubular cell injury^5^. In wild-type mice, the expression of *Abcc6* decreases 2 days after RAKI and normalized thereafter. Further studies, including organ-specific deletion of *Abcc6* will have to decipher how *Abcc6* deletion protect kidneys from RAKI (for example, modulation of the purinergic signal in the kidneys, immunomodulation, epithelial resistance to injury).

Interestingly, *Abcc6* deficiency led to protection against both acute and chronic rhabdomyolysis-induced kidney injuries. At day 2, FACS analyses showed a similar kidney immune cell distribution suggesting that additional non-immune mechanisms prevented RAKI at early stages. On the other hand, at month 2, mice with *Abcc6* deletion had lesser kidney fibrosis and displayed a significantly different kidney immune cell distribution then in *wt* mice (less CD3^+^ T-cells, more B220^+^ B-cells and a shift from R1 to R2 macrophages compared to *wt* mice) suggesting immune modulation and fibrosis prevention. In this study, we were unable to accurately assess the R2 sub-population that arose in *Abcc6*^-/-^ mice after kidney injury. Since M2 macrophages (mainly represented in the R2 population) are not a single cell population but include anti-inflammatory cells (that prevent excessive kidney damage), as well as extra-cellular matrix-producing cells (which promote fibrosis), it will be important to better decipher the respective role of each macrophage sub-populations in the context of ABCC6 inhibition.

Previous studies using immunohistochemistry reported that *Abcc6* is expressed in circulating leukocytes and some node lymphocytes^14^, suggesting that ABCC6 may have direct effect on immune cell activation or polarization. Indeed, we performed qPCR of FACS-sorted CD45^+^ immune cells extracted from kidneys and identified *Abcc6* transcripts in these cells. Alternatively, baseline liver ABCC6 deficiency and subsequent changes in systemic metabolism may increase the ability of the kidney to resist to injury. These findings deserve further attention.

The lack of a specific ABCC6 inhibitor currently precludes the confirmation of our data by a pharmacological approach but our findings now point to ABCC6 as a new potential molecular target to prevent RAKI and its consequences on deterioration of kidney function in the long term. Because chronic *ABCC6* deficiency leads to *pseudoxanthoma elasticum* and urolithiasis in humans, further studies will also have to confirm the protective role of transient ABCC6 inhibition on kidney dysfunction.

In summary, phosphate deposits in the kidney are frequent in RAKI but hydration with sodium bicarbonate or sodium chloride does not modify their extent and the kidney outcome. Surprisingly, blocking the ABCC6 transporter emerges as a new option to prevent RAKI and subsequent transition toward kidney fibrosis.

### Supplementary Information

Supplementary Figures.

## Data Availability

The datasets used and/or analysed during the current study are available from the corresponding author on reasonable request. No sequencing was performed.

## Abbreviations

BUN: Blood urea nitrogen
CKD: Chronic kidney disease
PPi: Inorganic pyrophosphate
KDIGO: Kidney diseases—improving global outcome
RAKI: Rhabdomyolysis-induced acute kidney injury
