# Supplementary material for: Abcc6 deficiency prevents rhabdomyolysis-induced acute kidney injury
Source: Sci Rep. 2023 Dec 6;13:21513. doi: 10.1038/s41598-023-47894-z (PMC10700332; doi:10.1038/s41598-023-47894-z)

**Supplementary Figure 1**. **Expression of *Abcc6*/*Mrp6*, as well as proximal tubule (*Aqp1*, *Lrp2*, *Cubn*) and immune (*F480*, *Cd11b*, *Cd3*) markers in FACS-sorted kidney cells (relative to *Hprt* expression)**. KAP2-iCre male mice also harboring a GFP (green fluorescent protein) gene flanked by LoxP sequences were sacrificed at weeks 12. **A.** Green fluorescence of the proximal tubule cells was confirmed by assessing the spontaneous green fluorescence of proximal tubules on kidney slices**. B.** Kidney cells of these male mice were FACS-sorted expression of *Abcc6* and other genes quantified in GFP^+^ cells (i.e., proximal tubule) and in GFP^-^CD45+ (i.e., immune) cells. **C.** Expression of Abcc6 was observed in both immune and proximal tubule cells, with a higher expression in the former.

1.
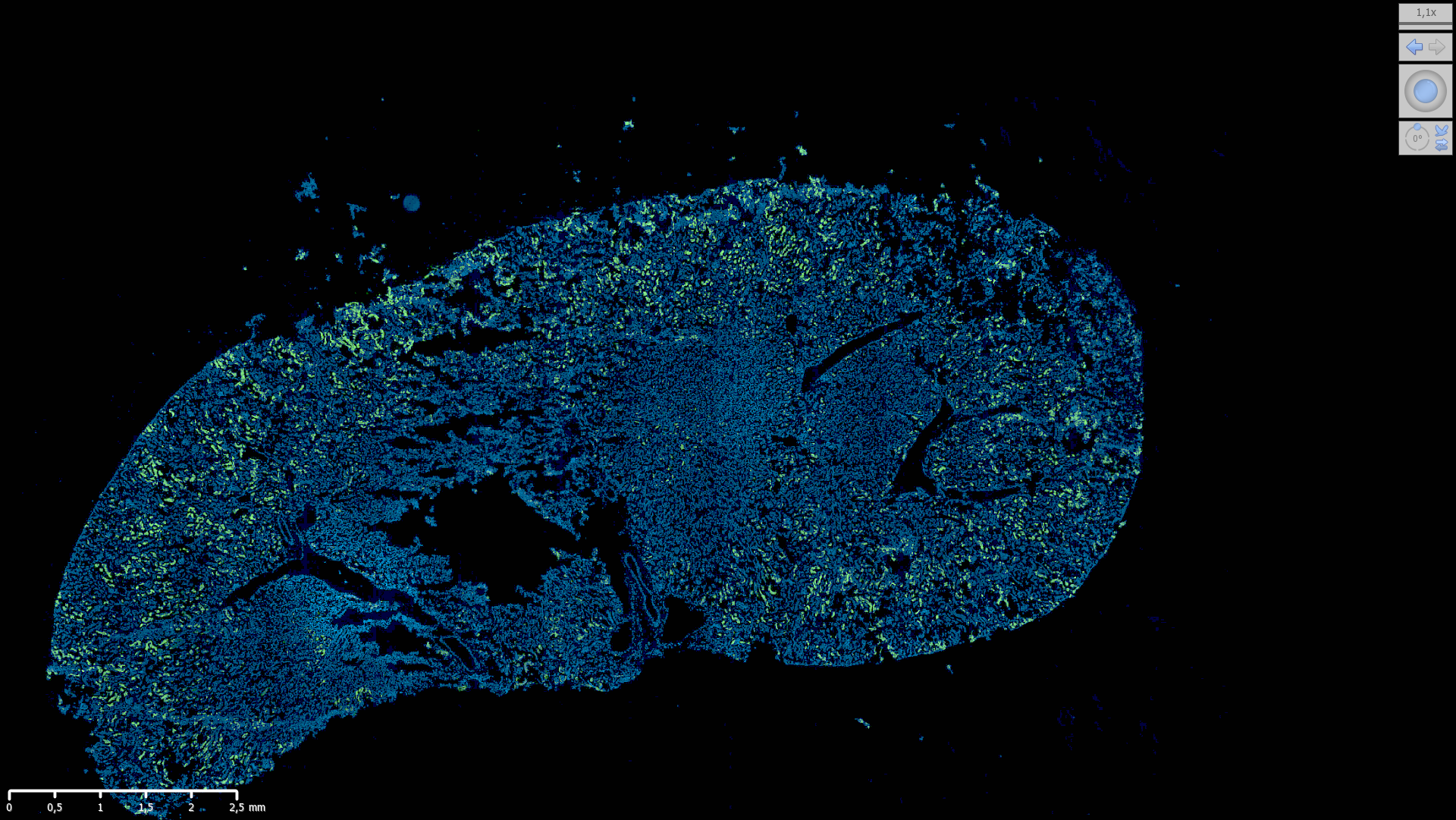

2.
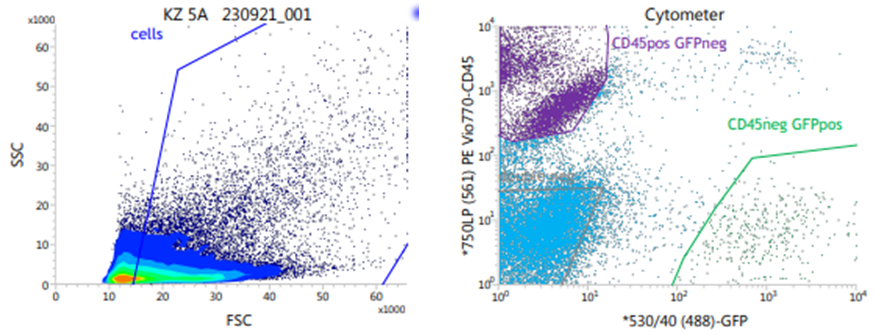

3.

**Supplementary Figure 2. Kidney expression of *Abcc6* (mRNA) before and 2 and 20 days after rhabdomyolysis**. Comparisons were made with ANOVA test and Dunnett post-correction (n=6 mice per group).

**Supplementary Figure 3.** Representative images of calcium phosphorus deposits within kidneys (Von Kossa staining) of *wt* and *Abcc6*^-/-^ mice at day 2 of RAKI and in sham.


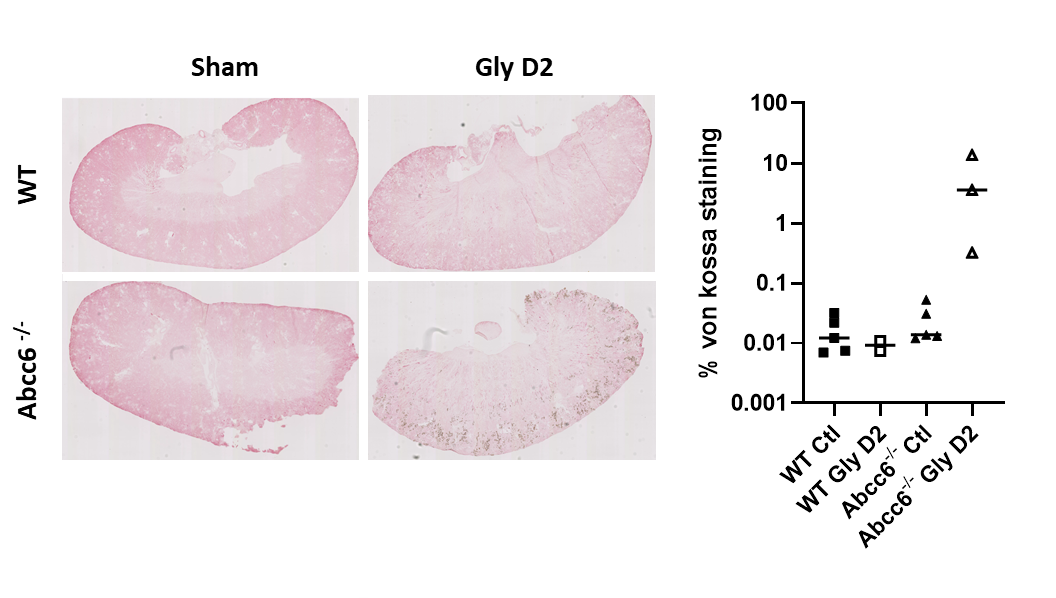

Supplement: Supplementary file 1 — Supplementary Figures. [file 41598_2023_47894_MOESM1_ESM.docx]
